# Giant gluteal lipoma-like liposarcoma: a case report

**DOI:** 10.1186/1477-7819-6-81

**Published:** 2008-07-29

**Authors:** Maitham Sultan, Hisham Burezq, Rameshwar L Bang, Moustafa El-Kabany, Waddah Eskaf

**Affiliations:** 1 Plastic and Reconstructive Surgery, Al-Babtain Center for Burns and Plastic Surgery, Sabah Health area, Ibn-Sina Hospital, PO Box 1574, Mishref, 40179, State of Kuwait; 2 Department of Surgery, Consultant Plastic and Reconstructive Surgeon, Faculty of Medicine, Kuwait University, State of Kuwait; 3 Department of Pathology, Kuwait Cancer Center, Sabah Health Area, State of Kuwait

## Abstract

**Background:**

Liposarcoma is the second most common soft tissue sarcoma in adults with a peak incidence between the 4^th ^and 6^th ^decade of life and slight preponderance to the male gender. It originates from multipotential primitive mesenchymal cells, rather than mature adipose tissue.

**Case presentation:**

An unusual case of a rapidly growing giant lipoma-like liposarcoma of the left gluteal and perineal areas in a young male was presented. The patient was managed by wide local excision of the lesion and coverage with split thickness skin graft. The key issues surrounding the treatment of lipoma-like liposarcoma and literature review is discussed.

**Conclusion:**

For such unusual case of this particular rapidly growing tumor, a longer follow-up is needed to evaluate the outcome in these cases.

## Background

Liposarcoma is the second most common soft tissue sarcoma in adults. This tumor originates from multipotential primitive mesenchymal cells rather than mature adipose tissue [1]. It commonly arises from extremities, particularly thighs, retroperitoneum, inguinal and paratesticular regions [2,3]. Chest wall, breast, mediastinum, small intestine, omentum and mesentery may also be involved. The peak age incidence of well differentiated, dedifferentiated, and pleomorphic liposarcoma occurs between the 4^th ^and 6^th ^decade of life with slight preponderance to the male gender[1]. The authors described an unusual case of a rapidly growing giant lipoma-like liposarcoma of the left gluteal region in a young adult patient. To the best of our knowledge, no such case is reported in the English literature.

## Case presentation

**Figure 1:**
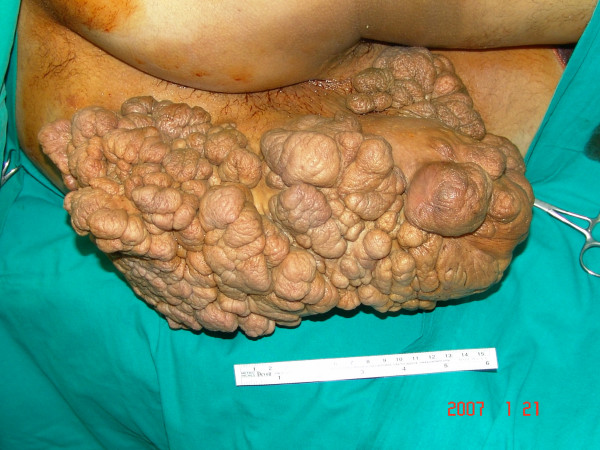
**Intra-operative photo showing a large polypoidal mass involving most of the left gluteal and perianal area**.

A 26 year old gentleman presented to our out-patient clinic at Al-Babtain Center for burns and plastic surgery with a 16 month history of a rapidly growing mass in the left gluteal region. Although this mass was interfering significantly with his daily normal activities, walking, anal hygiene and even with defecation, the patient did not search for treatment until that date when it became unbearable. Clinical examination revealed a huge well defined, polypoidal, cutaneous, fleshy mass of about 59 cm × 39 cm × 19 cm occupying most of the left gluteal and perianal area (Figure 1). The lesion had a narrower pedicle of about 20 cm × 30 cm. firmly attached to the underlying subcutaneous tissues. There were areas of peripheral necrosis associated with multiple patches of ulceration and foul odor. No pulsations or clinical thrill were identified. The systemic clinical examination was within normal limits. Hematological and biochemical work-up including CBC, renal function, liver function and coagulation profile all showed normal results.

**Figure 2:**
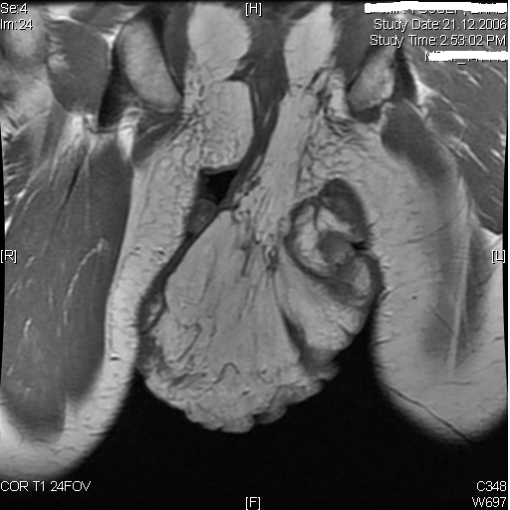
**T1-MRI axial section showing fatty signal intensity with evidence of solid component that showed moderate enhancement suggestive of liposarcoma**.

**Figure 3:**
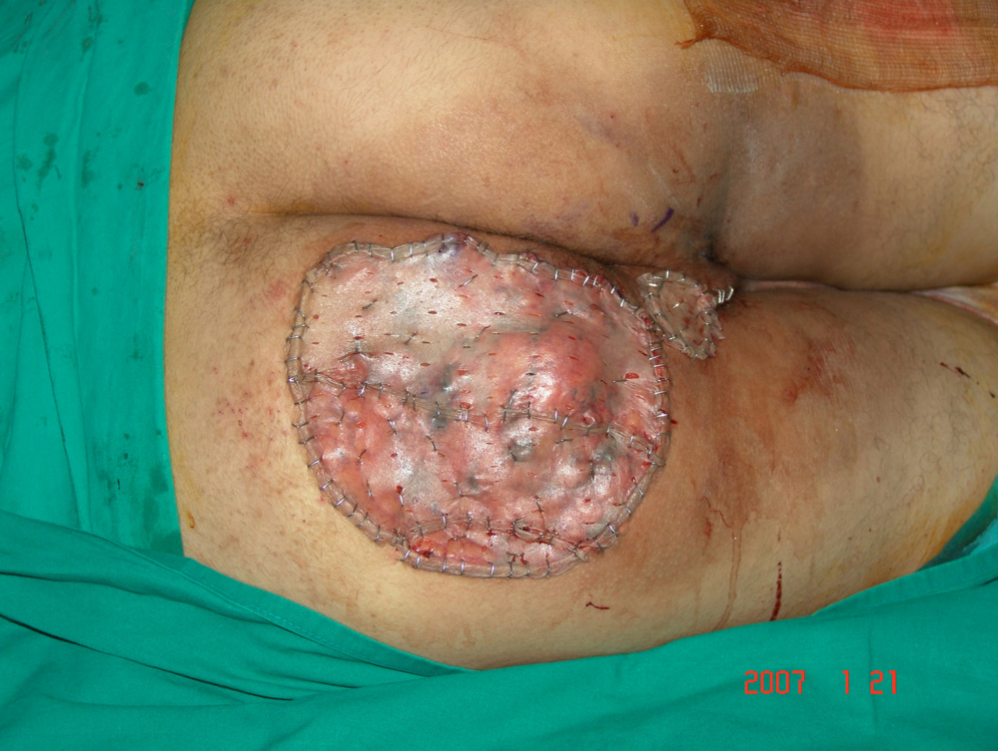
**Intra-operative photo showing the defect created after total resection and split thickness meshed skin grafting**.

Contrast enhanced MRI showed a huge irregular lobulated mass at the anal region extending outwards and inferiorly preserving the anal canal. The mass was mainly of fatty signal intensity with evidence of solid component that showed moderate enhancement suggestive of liposarcoma (figure 2). Incisional biopsy was performed to establish a tissue diagnosis that primarily revealed histological features of lipofibroma of benign nature. Because of the huge size and the rapid growth of the lesion and the possibility of missing the diagnosis with our incisional biopsy; the decision was taken to completely excise the lesion with 1 cm free margin down to the sub-fascial plane above the gluteal muscles. The excised specimen weighed 2615 grams with subsequent surgical defect about 22 cm × 27 cm which was reconstructed with a split thickness skin graft (Figure 3).

**Figure 4:**
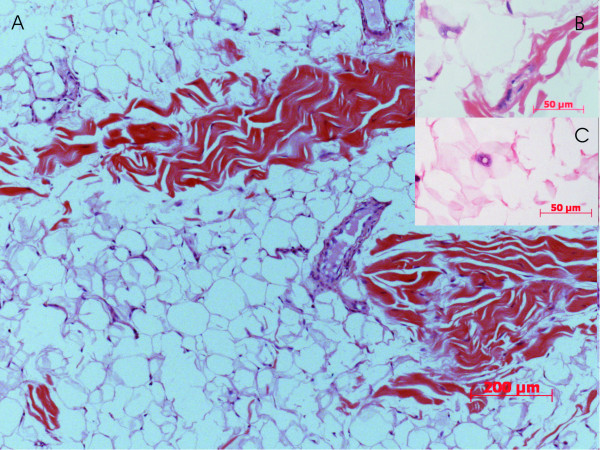
**Photomicrograph of excised lesion showing a well differentiated lipomatous tumor growth which exhibits at low magnification adipocyte with significant variation in size and shape (A) as well as occasional atypical adipocyte with enlarged hyperchromatic nuclei seen with high magnification (arrow)(B & C) (H&E stained, 5 u thick paraffin sections)**.

Surprisingly, the final pathology described yellow to white firm serial section with occasional necrotic foci. Histological sections showed variable sized lobules separated by fibrous septa. The lobules were composed of relatively mature adipocytic proliferation with significant variation in cell size, with occasional atypical lipoblasts exhibiting evidence of nuclear atypia and hyperchromasia. Hyperchromatic stromal cells were also, identified in the thickened fibrous bands. Monovacuolated and multivacuolated lipoblasts as well mononuclear chronic inflammatory elements were frequently seen (figure 4). Surface cutaneous tiny ulcerations were noticed and were partially replaced by inflammatory granulation tissue. Surgical clearance was adequate.

Post-operative CT scan of the abdomen and pelvis with oral and IV contrast were done as part of the metastatic workup and showed no abnormalities.

The case was discussed in the tumor board meeting and the decision was made to achieve an adequate loco-regional control by wide local excise. The operation was done in a left lateral decubitus position under general anesthesia with oro-tracheal intubation. Total excision of the lesion was done with a 2 cm free margin down to a deep sub-facial plane exposing the gluteal maximus muscle which was found to be free of the disease. The resultant soft tissue defect was covered with a split thickness skin graft taken from the left thigh. Part of the graft was lost because of a localized pseudomonas infection which healed completely with daily wound care and without surgical intervention. The final histopathology diagnosis was well-differentiated and well defined completely excised lipoma-like liposarcoma, therefore adjuvant radiotherapy was not indicated. The patient was followed for about 16 months showing no evidence of recurrence.

## Discussion

Adipocytic tumors represent the largest single group of mesenchymal neoplasms, due to the high prevalence of lipomas and their variants. Liposarcoma represents the single most common soft tissue sarcoma in adults, accounting for approximately 20% of all cases[4]. Its principal histological subtypes; well differentiated, myxoid/round cell and pleomorphic are entirely separate diseases with different morphology, genetics and natural history. The principal changes in the recent WHO classification demonstrates that atypical lipomatous tumors and well differentiated liposarcoma are essentially synonymous and that site-specific variations in behavior relate only to surgical resectability [5].

This male patient was quite young though the peak incidence is considered between the 4^th ^and 6^th ^decade of life[1]. The presentation was due to the discomfort in maintaining the day today activities and foul odor it emitted. The lesion was a cutaneous outward growth and it attained considerable size in a short period of time and presented a difficult dilemma for the diagnosis.

Liposarcomas can be divided into three basic histological categories; well-differentiated liposarcomas which morphologically subdivided into lipoma-like, sclerosing, inflammatory or spindle cell type, myxoid liposarcoma and pleomorphic liposarcoma [5].

Well-differentiated liposarcomas account for about 40%–45% of all liposarcoma and therefore represent the larger subgroup of adipocytic malignancies. Although the recurrence rate can reach up to 30% of the cases, this tumor which is surgically amenable behaves as a benign neoplasm and is not known to metastasize, thus requiring a less aggressive treatment [6]. The most important prognostic factor for well-differentiated liposarcoma is its anatomic location where superficial lesions are considered favorable while deeply seated lesions such as retroperitoneal or mediastinal liposarcoma are associated with increased recurrence and metastatic rates [1,6].

The benefit of wide local excision over marginal excision is recognized in the literature [7]. In our case, we have excised the lesion with 1 cm margin down to a subfascial plane over the gluteal muscles to have good local control. We could not find any evidence in the literature suggesting a benefit in outcome with the use of postoperative radiotherapy. Some authors caution against its use to treat this lesion due to the uncertainty about its role in the dedifferentiation process ^4^.

## Conclusion

An unusual case of a rapidly growing, giant gluteal lipoma-like liposarcoma was presented. Surgical excision is the main treatment for most primary soft tissue sarcomas. As such every effort should be made to achieve complete tumor resection. A longer follow-up is needed to evaluate the outcome such cases. Although not used in this case, we wonder if radiotherapy could be used in such giant tumors to improve the loco-regional control.

## Competing interests

The authors declare that they have no competing interests.

## Authors' contributions

HB Substantial contributions to conception, design, and in drafting the manuscript or revising it critically for important intellectual content. MS Substantial contribution in literature review and data analysis. RLB Substantial contributions in reviewing the draft and the addition of important data to the text. WE analysis of slides taken from the patient and reaching a diagnosis. ME analyzed the data related to pathology in the text with a significant contribution in drafting. All authors read and approved the final manuscript.
